# Role of the repartition of wetland breeding sites on the spatial distribution of *Anopheles *and *Culex*, human disease vectors in Southern France

**DOI:** 10.1186/1756-3305-4-65

**Published:** 2011-05-06

**Authors:** Priscilla Cailly, Thomas Balenghien, Pauline Ezanno, Didier Fontenille, Céline Toty, Annelise Tran

**Affiliations:** 1 INRA, ONIRIS, UMR1300 Bio-agression, Epidémiologie et Analyse de Risques, Nantes, France; 2 CIRAD, UPR AGIRs Animal et Gestion Intégrée des Risques, UMR TETIS, Territoires, Environnement, Télédétection et Information Spatiale, Montpellier, France; 3 CIRAD, UMR Contrôle des maladies, Montpellier, France; 4 IRD, MIVEGEC Maladies Infectieuses et Vecteurs: Ecologie, Génétique, Evolution et Contrôle, Montpellier, France; 5 Centre de Recherche et de Veille sur les maladies émergentes dans l'Océan Indien, Sainte Clotilde, Ile de la Réunion

## Abstract

**Background:**

In this study, carried out in the Camargue region (France), we combined entomological data with geomatic and modelling tools to assess whether the location of breeding sites may explain the spatial distribution of adult mosquitoes. The species studied are important and competent disease vectors in Europe: *Culex modestus *Ficalbi and *Cx. pipiens *Linnaeus (West Nile virus), *Anopheles atroparvus *Van Thiel, a former *Plasmodium *vector, and *An. melanoon *Hackett, competent to transmit *Plasmodium*.

Using a logistic regression model, we first evaluated which land cover variables determined the presence of *Culex *and *Anopheles *larva. The resulting probability map of larval presence then was used to project the average probability of finding adults in a buffer area. This was compared to the actual number of adults collected, providing a quantitative assessment of adult dispersal ability for each species.

**Results:**

The distribution of *Cx. modestus *and *An. melanoon *is mainly driven by the repartition of irrigated farm fields and reed beds, their specific breeding habitats. The presence of breeding sites explained the distribution of adults of both species. The buffer size, reflecting the adult dispersal ability, was 700 m for *Cx. modestus *and 1000 m for *An. melanoon*. The comparatively stronger correlation observed for *Cx. modestus *suggested that other factors may affect the distribution of adult *An. melanoon*. We did not find any association between *Cx. pipiens *larval presence and the biotope due to the species' ubiquist character.

**Conclusion:**

By applying the same method to different species, we highlighted different strengths of association between land cover (irrigated farm fields and reed beds), larval presence and adult population distribution.

This paper demonstrates the power of geomatic tools to quantify the spatial organization of mosquito populations, and allows a better understanding of links between landcover, breeding habitats, presence of immature mosquito populations and adult distributions for different species.

## Background

Environmental conditions may determine the presence of species and their population dynamics, especially for insects which are highly dependent on climatic conditions and landscape organization [1-4]. Geographical Information Systems (GIS) and spatial analyses are used widely in ecology [4-8] to understand the relation between habitats and the presence or abundance of species. When applied to pathogen vectors, these tools render it possible to develop control strategies and compute risk maps [8-12].

For mosquitoes, which are the most important vectors of human pathogens (malarial *Plasmodium *species, dengue, Chikungunya, yellow fever viruses, *etc*.), several authors have noted the possibility of mapping the distribution of immature and adult populations as a function of landscape characteristics [13-16]. Mosquito larvae are purely aquatic and develop in water bodies, the type of which is more or less specific to each species. Various landscape components, such as land cover, hydrologic networks, vegetation characteristics, and human and animal population distributions, may determine the presence and abundance of immature mosquitoes, the dispersion of adult from breeding sites, and the abundance of adult mosquitoes in different habitats. Human activities that modify the landscape, such as irrigated fields and land settlements, consequently may impact the temporal and spatial distribution of mosquitoes [8,10], *e.g*. change the availability of breeding sites in time and space.

The spatial distribution of mosquitoes determines their contact with vertebrate hosts, which influences in turn the spatial spread of vector-borne pathogens. An understanding of the relationship between the availability of productive breeding sites and landscape and climatic conditions, and of how mosquitoes disperse from breeding sites, should therefore contribute to more accurate predictions of disease spread.

Tran *et al. *(2008) [16] proposed a framework to build a predictive environmental model for immature and adult mosquito distribution that analyzes the relationship between landscape characteristics and mosquito field collections (larvae and adults). They successfully applied this approach on *Anopheles hyrcanus *(Pallas), a potential malaria vector, in the Camargue region of southern France. They demonstrated that the repartition of this species' breeding sites was the main driver behind the distribution of the adult population. In this paper, we aimed to evaluate the impact of the repartition of wetland breeding sites on the spatial distribution of *Culex modestus *Ficalbi, *Cx. pipiens *Linnaeus, and *An. maculipennis *Complex. For the latter, we focussed on *An. atroparvus *Van Thiel, a former plasmodium vector in southern France, and *An. melanoon *Hackett, which is competent to transmit malaria parasites, although its preference for large mammal hosts may limit its potential role as a vector [17-20]. Both *Culex *species are efficient and active vectors of the West Nile virus (WNV) in the Camargue region [21-24]. We applied the same methodology developed by Tran *et al. *(2008) [16] on *An. hyrcanus *on *An. melanoon, Cx. modestus *and *Cx. pipiens*. The results for the four species consequently can be compared and discussed.

## Methods

### Study area

**Figure 1:**
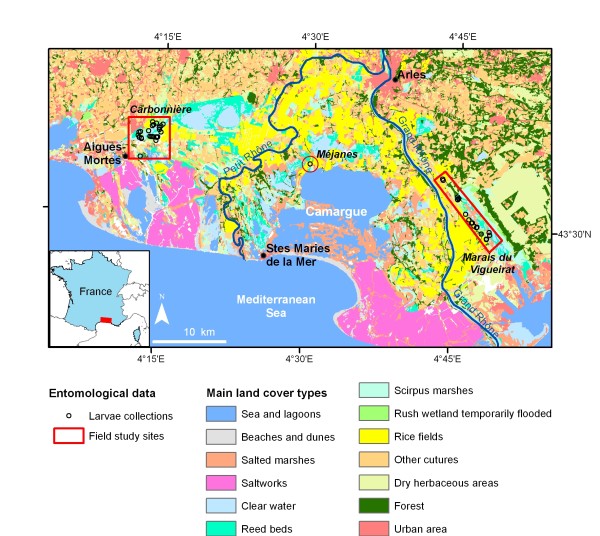
**Location of the study area and the mosquito sampling sites in the Camargue, Southern France**. Background: Land cover map derived from Landsat ETM+ imagery.

The Camargue is a region located in the Rhône River delta in southern France (between 43.33° and 43.73° north and 4.05° and 4.93° east) (Figure 1). This area has a Mediterranean climate characterized by warm, dry summers and mild, wet winters. Sparsely populated by humans, the region is an ecological mosaic shaped by the sea in the south and agricultural activities in the north. The southern landscapes are influenced by the presence of saltwater, and are composed of associations of halophytic plants and salt ponds. During the summer, evaporation leads to a water deficit that is compensated by artificial flooding linked to human activities (cultivation, extensive breeding of bulls and horses, hunting and fishing). Vegetation associated with fresh water is composed of reed marshes, wet meadows, and riverine forest. The northern landscapes of this area are mostly composed of rice fields and pastures.

Larva and adult mosquitoes were collected on three sites that are potentially suitable for *Anopheles *and *Culex *species (Figure 1). The "Carbonnière" site includes arable paddies and different types of marshes. There is a considerable amount of human activity through agriculture, animal husbandry, hunting, and tourism. Mosquito control is carried out almost exclusively against the pest species *Aedes caspius *(Pallas) and *Ae. detritus *Haliday, and mainly is applied to their principal breeding sites: salt marshes and irrigation canals. Mosquito control activities therefore have only limited consequences on *Culex *and *Anopheles *populations. Moreover, insecticide sprayings on rice fields, which previously had affected mosquito populations, currently are restricted largely due to European regulations [18]. The "Marais du Vigueirat" site is a nature reserve holding a large wetland with marshes and reed beds. Human activities and impacts in the reserve are limited, and mosquito populations are not controlled. The "Méjanes" site is a rice paddy area located inside a nature reserve where mosquito populations are not controlled.

### Entomological data

To collect larvae, 80 potential breeding sites (37 in "Marais du Vigueirat", 41 in "Carbonnière" and 2 in "Méjanes") were visited in 2006 every month from April to October, the period when mosquitoes are active. Standard dipping techniques were used [25]; larvae were stored in absolute alcohol and identified down to the species level using a morphological identification key [26] and Polymerase Chain Reaction (PCR) assay to separate *An. atroparvus *and *An. melanoon *[27]. We organized the larval collection data by presence (at least one larva collected during the whole period) and absence (no larva collected). The larvae of five *Culex *and *Anopheles *species were collected: *Cx. modestus, Cx. pipiens, An. atroparvus, An. melanoon*, and *An. hyrcanus*. All five species were present on all three sites with the exception of *An. atroparvus*; only a few larvae (N = 3) of this species were collected. Data on *An. hyrcanus *were analyzed in a previous paper [16]. We therefore focused our statistical analysis on the three other abundant species: *Cx. modestus, Cx. pipiens*, and *An. melanoon*.

Adult mosquitoes were captured the previous year, 2005, from March to October. As numerous individuals working in the study area reported that environmental conditions and mosquito populations were similar in 2005 and 2006, there is no reason to believe that the gap between the collection of adults and larvae prejudiced the study. Adults were captured on 8 locations in "Marais du Vigueirat" and 8 locations in "Carbonnière". Centers for Disease Control (CDC) light traps associated with carbon dioxide dry ice were used from 19:00 to 10:00 the following morning for two consecutive nights once every two weeks (512 collections). Adults then were identified down to the species level using the same techniques applied to larvae. In 2005, we collected adults of fours species, *Cx. modestus, Cx. pipiens, An. melanoon *and *An. hyrcanus*, on the two field study sites. Due to logistical constraints, it was not possible to carry out collections of adult mosquitoes in "Méjanes"; the site was added in 2006 to catch additional larvae.

All collections of adult and larva mosquitoes were localized using a Global Positioning System (GPS) receiver.

### Land cover map

A land cover map was obtained using a supervised object-oriented classification of two Landsat Enhanced Thematic Mapper (ETM+; spatial resolution of 30 m) images from the dry (July 21, 2001) and wet (October 25, 2001) seasons (method presented in [16]). This map was computed to include the main wetland mosquito habitats: rice fields, reed beds, *Scirpus *marshes, temporarily flooded rush wetland, and clear water (Figure 1).

### Spatial distribution of mosquito larvae

We used a logistic regression to test the association between the presence of larva in the breeding sites (response variable) and the land cover classes (explanatory qualitative variables) to estimate the regional risk of larval presence. Logistic regression commonly is used to study the relationship between a binary variable (presence/absence) and risk factors which may be qualitative or quantitative variables [28]. First, spatial autocorrelations of larval samples were tested (calculation of Moran's I index) and three logistic regression models were built [16]. The model that had the best accuracy then was chosen. We used a multi-cross-validation to assess the stability and quality of the predictions of the model implemented with R freeware [29]. The sample (n = 80) first was divided randomly into two sub-samples. One sub-sample (n_1 _= 60) was used to build the logistic regression model. Larval presence then was predicted using a risk threshold value of 0.5, which was the optimal cut-off threshold estimated by a Receiver-Operating Characteristic analysis [30]. The model accuracy (overall accuracy, sensitivity (confidence interval 95%) and specificity (confidence interval 95%)) was assessed by comparing the predicted values and the values of the second sub-sample (n_2 _= 20). This procedure was repeated 1,000 times to assess the stability of the model and determine its parameters. Finally, for each species, we calculated a larval index for all map pixels using ESRI ArcGIS™ (Spatial Analyst Tools). This larval index was the probability of larva being present at least once in the year and was obtained by applying the logistic transformation to each pixel according to the pixel's land cover class. As mosquito control measures were being implemented during the capture period, they were taken into account in the larval index maps (*e.g*. rice fields likely to be treated were excluded).

### Spatial distribution of adult mosquitoes

Assuming that the presence of breeding sites in the environment impacts the distribution of adult mosquito populations, we assessed the association between the probability of larval presence, estimated by the larval index, and the maximum adult mosquito catch. We compared the adult index, which was the projected probability of adult presence based on the larval index, with the maximum number of adults captured during the year. The risk of pathogen transmission increases with host/vector contacts, which in turn increase with the number of vectors. Tran *et al. *(2008) [16] and Ponçon *et al. *(2008) [20] used this maximum adult mosquito catch value to assess the highest risk of host/vector contact in the Camargue. For each adult collection site, we used ArcGIS™ to compute an adult index as the average of the larval index in a buffer area around the trapping site. Different buffer sizes were tested (ranging from 100 to 3,000 meters) to test for a large range of dispersion radiuses. We calculated the Pearson correlation coefficient between the computed adult index and the observed adult abundance. The optimum buffer size was the smallest one maximising the correlation.

## Results

### Mosquito collections

**Figure 2:**
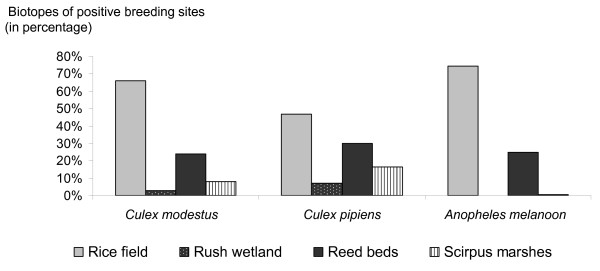
**Contribution of each biotope to the breeding sites of mosquito species in the Camargue, France**. Larvae were captured from March to October 2005. Larvae captured: 144 for *An. melanoon*, 328 for *Cx. pipiens*, 428 *Cx. modestus*.

For *Cx. modestus, Cx. pipiens *and *An. melanoon*, the most frequented breeding sites were rice fields followed by reed beds (Figure 2). Only *Culex *larvae were collected in rush wetlands. All three species were collected in *Scirpus *marshes.

Populations were more abundant in "Marais du Vigueirat" than "Carbonnière": 163,922 *Cx. modestus*, 60,944 *Cx. pipiens *and 1,290 *An. melanoon *were collected in "Marais du Vigueirat" compared to 26,279 *Cx. modestus*, 30,626 *Cx. pipiens *and 241 *An. melanoon *adults collected in "Carbonnière".

### Spatial distribution of larval populations

Larval abundances were not spatially auto-correlated (Moran's I indices = 0.07, -0.03, 0.17 and associated p-values = 0.45, 0.89, 0.07 for *An. melanoon, Cx. modestus *and *Cx. pipiens *respectively). Therefore, observations could be considered as being independent in the analysis. The model with the best compromise between sensitivity and specificity, as well as the best overall accuracy, explained the presence of larvae as a function of biotope (for *Cx. modestus *sensitivity = 0.57[0.33-0.83], specificity = 0.87[0.73-1], overall accuracy = 0.75[0.58-0.89]; for *Cx. pipiens *sensitivity = 0.33 [0-0.67], specificity = 0.7 [0.62-0.76], overall accuracy = 0.69[0.53-084]; for *An. melanoon *sensitivity = 0.75[0.4-1], specificity = 0.83[0.71-0.93], overall accuracy = 0.79[0.63-0.89]). Rice field and reed bed biotopes were associated with the presence of *Cx. modestus *and *An. melanoon *larvae (Table 1). For *Cx. pipiens*, we did not found any significant association.

**Table 1:** Prediction of larval presence using regression models in the Camargue region, France

Species	Model	Regression coefficient	[95%CI^a^]	p^b^
*Culex modestus*	Intercept	-17.09	[-17.57;-16.67]	
	Biotope			
	Rush wetland	12.74	[-4.19E-09;16.47]	
	Rice field	17.93	[17.02;19.11]	*
	Reed beds	16.12	[15.34;17.01]	*
	Marshes with *Scirpus*	15.97	[14.96;16.87]	
*Culex pipiens*	Intercept	-17.61	[-18.57;-17.57]	
	Biotope			
	Rush wetland	5.93E-11	[-7.65E-09;7.66E-09]	
	Rice field	17.45	[17.02;18.11]	
	Reed beds	16.59	[16.01;17.01]	
	Marshes with *Scirpus*	15.95	[15.17;16.47]	
*Anopheles melanoon*	Intercept	-19.57	[-19.57;-19.57]	**
	Biotope			
	Rush wetland	-5.54E-11	[-3.91E-08;3.47E-08]	
	Rice field	19.91	[19.48;20.34]	**
	Reed beds	17.93	[17.21;18.29]	**
	Marshes with *Scirpus*	-5.54E-11	[-3.91E-08;3.47E-08]	

^a^CI: confidence interval; ^b^p: p < 0.01 (**); p < 0.05 (*)

**Figure 3:**
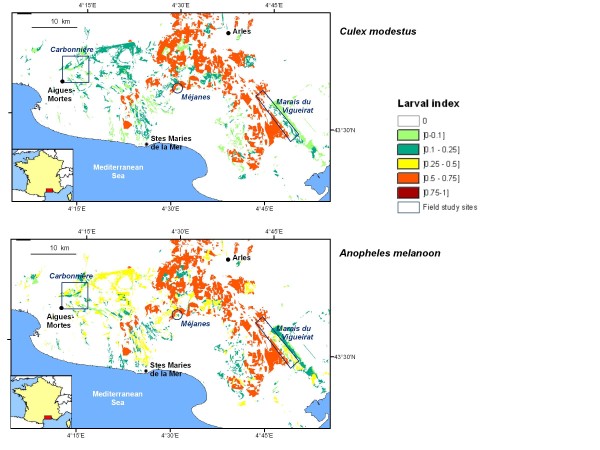
**Maps of the larval index for two mosquito species in the Camargue, France**. Colour scale of the probability of larval presence ranges from green (low probability) to brown (high probability).

From these results, larval index maps were obtained (Figure 3) that show the spatial repartition of *Cx. modestus *and *An. melanoon *larvae over the entire landscape.

### Spatial distribution of adult populations

**Figure 4:**
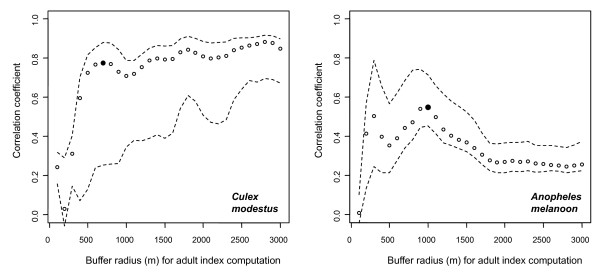
**Correlation the estimated and the observed mosquito adult abundance, function of the buffer radius, Camargue, France**. In full black dot, the buffer radius identified as the optimal buffer size. Dashed lines indicate the confidence envelope, computed by leave-one-out method.

Adult indices were significantly correlated with the observed abundance of *Cx. modestus *and *An. melanoon*. For *Cx. modestus*, the correlation coefficient between the computed adult index and the observed abundance increased with the buffer radius up to 700 m, when the correlation coefficient reached a plateau (Figure 4). For *An. melanoon*, this coefficient increased with the buffer radius up to 1000 m, and then decreased (Figure 4). The correlation was stronger for *Cx. modestus *(Pearson r = 0.78 for buffer radius = 700 m, p < 0.01) than for *An. melanoon *(Pearson r = 0.55 for buffer radius = 1000 m, p < 0.05).

## Discussion

Models of mosquito spatio-temporal dynamics can be used to focus surveillance, assess control strategies, and understand mechanisms of the epidemiology of mosquito-borne diseases. However, it remains difficult to determine the environmental factors structuring the spatial distribution of mosquito populations on a large scale [31]. In this study, we used landscape characteristics and mosquito field collections (larvae and adults) to produce predictive maps of the fine spatial distribution of populations of Cx. *modestus *and *An. melanoon *larvae and to assess the dispersion ability of adults in a wetland area of southern France.

We confirmed that *Cx. modestus *and *An. melanoon *larvae are present mainly in irrigated farm fields, as was demonstrated in a previous entomological survey in this area [32]. Irrigated farmland such as rice fields are known to be important breeding sites for *Anopheles *species [16,33-35]. Other biotopes such as reed beds are less productive; however, they may constitute the only available breeding sites when rice fields are dry [32,36]. The wide distribution of *Cx. modestus *larvae can be explained by their ability to colonize relatively salty breeding sites such as *Scirpus *marshes. In contrast, *Cx. pipiens *larval presence was not related to a preferred biotope among the sampled wetland habitats. This result confirms their ability to colonize diverse breeding sites, rendering the species ubiquitous [3,32].

The adult index was correlated with the observed abundance of *Cx. modestus *and *An. melanoon*. The spatial distribution of the breeding sites thus strongly influenced adult abundance, as was suggested by studies on other *Anopheles *species [8,9,37]. This correlation was lower for *An. melanoon *than for *Cx. modestus*, suggesting that other factors may influence *An. melanoon *distribution. Dispersion occurs when mosquitoes are seeking hosts, resting places and breeding sites. Host presence therefore may be an explanatory factor of the observed adult abundance [9,37] because the traps mainly collected host-seeking females. Moreover, vegetation characteristics such as openness and topography are known to drive female dispersion [8,33]. However, the study area is flat, with no elevations. Although wind is known to inhibit the flight activity of mosquitoes [3], it also may transport mosquitoes over long distances. We therefore do not think that wind plays an important role in structuring mosquito populations in the Camargue. Different optimum buffer sizes, which can be interpreted as different distances of active dispersion around an emergence site, were observed for the two species, the smallest being observed for *Cx. modestus*. This species is known to have limited dispersion ability [38]. We estimated the average active dispersion distance of *An. melanoon *to be one kilometre, which is consistent with findings in the literature [3,33]. This dispersion range should not only be regarded as an individual flight range, but as the average dispersion ability of the population.

By applying the same method to different species, *An. hyrcanus *(results presented in [16]), *An. melanoon, Cx. modestus *and *Cx. pipiens *(this study), it was possible to highlight different strengths of association between land cover, larval presence and adult population distributions. The adult index computed for *An. hyrcanus *was correlated closely with the observed adult abundance values (Pearson r = 0.97, buffer size = 300 m, p < 0.05). All together, the results indicate that these associations are stronger for species with marked preferences for breeding habitats and more restricted active dispersion ability. When mosquito species are opportunist, they can choose between a range of potential hosts; the presence and abundance of one particular host consequently has less influence on their distribution. Moreover, we illustrated the capacity of spatial analyses to characterize important features of mosquito behaviour (such as breeding site preferences) and to quantify some of them (such as dispersion distance). Based on our results, predictive maps of the presence of *Cx. modestus *and *An. melanoon *could be produced. However, quantitative maps accounting for seasonal variations in abundance could not be produced without the use of a predictive model of population dynamics.

The time gap between the acquisition of satellite data (2001) and the entomological surveys (2005-2006) was not prejudicial in a context of relative landscape stability [39]; this was confirmed during field studies [40]. Human activities in the Camargue, including non-agricultural areas and nature reserves, which might affect the landscape mainly consist of flooding for fishing, hunting, and conservation purposes. Inter-annual variations of climatic conditions therefore have limited consequences on landscape in the Camargue. The same sampling procedures were carried out on the "Marais du Vigueirat" and "Carbonnière" sites (8 traps). We demonstrated that we could relate the repartition of breeding sites to adult abundance. Differences in adult abundance thus appear to be mainly due to differences in the landscape of the sites.

The epidemiology of vector-borne diseases largely depends on vectors which are affected by environmental conditions. As these environmental conditions evolve in time and space, the study of the epidemiology of such diseases requires a combination of several approaches such as field entomology, geomatics, and modelling. Field entomology informs mosquito biology. Geomatics identifies links that exist between vectors and their environment. Modeling integrates all of this knowledge to better understand the functioning of the epidemiology of vector-borne diseases and to identify risk zones. Moreover, spatio-temporal models enable mosquito abundance over time and dispersion in space to be calculated by taking into account changes in the landscape [10]. This tool could be used to map the risk of disease emergence under different scenarios of climate and anthropogenic environmental change.

## Competing interests

The authors declare that they have no competing interests.

## Authors' contributions

PC carried out data analyses and drafted the manuscript. AT carried out the image processing and data analyses. TB and PE participated in the interpretation of results. CT and DF designed and carried out the entomological field work and identification of mosquito species. PC, AT, TB and PE wrote the paper and other co-authors commented on it. All authors read and approved the final manuscript.
